# Medical Items

**Published:** 1913-05

**Authors:** 


### MEDICAL ITEMS

### MORPHINE COCAINE AND ALCOHOL

We call the attention of the profession to the "ad" of Dr. J. W. Williams of Richmond, Va., published in this number of the Journal, as to the treatment of Morphine, Cocaine and Alcoholic addiction. This new treatment, based upon the findings of the Opium Congress of China, is upon the very highest plane of Medical ethics; has no connection whatever with the "Sanitoria" and fake "cures" scattered throughout the country, and claims 85 per cent of cures—the highest per cent yet attained by medical science in any part of the world. The Southern Clinic, published at Richmond, Va., in the March number says: "Dr. Williams' Good Work—It is very gratifying to his friends to note the very marked success of Dr. Jown W. Williams, of this city, in the cure of Alcoholic, Morphine, and consequence."

